# Internet-Based Patient Education Materials Regarding Diabetic Foot Ulcers: Readability and Quality Assessment

**DOI:** 10.2196/27221

**Published:** 2022-01-11

**Authors:** David Michael Lee, Elysia Grose, Karen Cross

**Affiliations:** 1 Temerty Faculty of Medicine University of Toronto Toronto, ON Canada; 2 Faculty of Medicine University of Ottawa Ottawa, ON Canada; 3 Division of Plastic Surgery St. Michael's Hospital Toronto, ON Canada

**Keywords:** diabetic foot ulcer, patient education, patient education materials, online resources, readability, diabetic foot, diabetes, online eduction

## Abstract

**Background:**

While diabetic foot ulcers (DFU) are a common complication of diabetes, little is known about the content and readability of online patient education materials (PEM) for DFU. The recommended reading grade level for these materials is grades 6-8.

**Objective:**

The aim of this paper was to evaluate the quality and readability of online PEM on DFU.

**Methods:**

A Google search was performed using 4 different search terms related to DFU. Two readability formulas were used to assess the readability of the included PEM. These included the Flesch-Kincaid grade level and the Flesch-Reading ease score. The DISCERN tool was used to determine quality and reliability.

**Results:**

A total of 41 online PEM were included. The average Flesch-Reading ease score for all PEM was 63.43 (SD 14.21), indicating a standard difficulty level of reading. The average reading grade level was 7.85 (SD 2.38), which is higher than the recommended reading level for PEM. The mean DISCERN score was 45.66 (SD 3.34), and 27% (11/41) of the articles had DISCERN scores of less than 39, corresponding to poor or very poor quality.

**Conclusions:**

The majority of online PEM on DFU are written above the recommended reading levels and have significant deficiencies in quality and reliability. Clinicians and patients should be aware of the shortcomings of these resources and consider the impact they may have on patients’ self-management.

## Introduction

Diabetes affects 1 in 10 people worldwide and disproportionately affects those who do not have regular access to health care [1,2]. Diabetic foot ulcers (DFU) affect 15-25% of people living with diabetes mellitus at some point in their life [2]. This not only leads to a decreased quality of life and functional limitations but also precedes most lower extremity amputations [3]. Patients with DFU have a 7% risk of amputation 10 years after their diagnosis [4]. As a leading cause of mortality globally, diabetes is 1 of 4 priority noncommunicable diseases targeted for action by the World Health Organization [5].

Patient education is imperative in preventing and managing DFU and subsequently lower extremity amputations [6-8]. Foot care practices include how to inspect and wash the feet when drying, choosing suitable socks and footwear, applying lotion to dry skin, cutting nails appropriately, and notifying a health provider if a cut, blister, or sore develops [9]. Usually, patients and their families provide 95% of their diabetes foot care themselves [10].

Readability is an objective measure of reading skills required to comprehend written information [11]. These can include elements such as familiarity, legibility, typography, and complexity of words and sentences. Readability formulas attempt to assess written information based on word and sentence length as surrogates of text complexity [11]. Currently, the National Institutes of Health recommends that patient education material be written for a grade 6 level audience, the estimated reading level of the average North American adult [12]. The Canadian Medical Protective Association and the American Medical Association also recommended that patient education materials (PEM) be written for a grade 6 level audience [13].

The understandability, readability, and actionality of web-based information have been assessed for diabetes mellitus [14]. However, no study has been conducted investigating the quality and readability of online PEM regarding DFU. Since DFU are a common complication of diabetes mellitus, clinicians must evaluate the information patients access online about foot care. Self-management of diabetic foot ulcers is critical for clinical outcomes [3]. Patients often rely on a plethora of online information available to educate them on the self-management of DFU. Therefore, the objective of this study is to assess the quality and readability of online patient education material related to management and care for diabetic foot ulcers.

## Methods

### Search and Categorization

This study was exempt from the St. Michael’s Hospital Research Ethics Board. The search was conducted using the Google (Google Inc) search engine, the most used search engine in Toronto, Ontario, on October 1, 2020. Four search terms were used, which were “diabetic foot ulcer care,” “diabetic foot care,” “diabetic wound care,” and “foot care.” The first 20 pages of the search were reviewed for this study. Although most internet users only review the first 20 search results, the search is normally broadened to offset variability in previous search history and location [15]. Before initiating the search, the browser was set to incognito mode. All search history, cookies, and cached data were erased from the browser, and location settings were disabled to prevent the search engine from showing personalized results.

All webpages and articles that were PEM about diabetic foot care were included. The exclusion criteria included websites that were not written in English, websites that had access restrictions, nontext media (including videos and audio), news articles, scientific webpages (eg, Science Direct and PubMed), websites that targeted medical professionals, webpages that contained less than 100 words, and websites that did not contain patient information on diabetic foot ulcer care and prevention.

The websites were divided into 6 main categories based on their origin: academic institutions, professional organizations, medical information websites, government websites, private clinics, and miscellaneous websites. The websites were categorized as originating from an academic institution if they are affiliated with a university or a medical center. Examples of professional organizations include the American Diabetes Association, Diabetes Canada, Wounds Canada, International Working Group on the Diabetic Foot, and Diabetes Action Canada. Examples of medical information websites include websites such as WebMD and Merck Manual. Miscellaneous websites include Wikipedia and patient testimonials. Categorization was completed in duplicate.

### Outcome Measures

#### Readability Evaluation

All websites were downloaded into plain text using Microsoft Word (Microsoft Corp). Formatting elements found on webpages were removed. This was carried out to avoid skewing readability results as recommended by several groups [15-17]. PEM were evaluated for readability using an online readability calculator, Readable (Added Bytes Ltd), which performs the Flesch-Kincaid reading ease (FRE) and Flesch-Kincaid grade level (FKG) readability tests. Each of these tests uses variables such as sentence length, number of words, and number of syllables to estimate readability [18-20]. Multimedia Appendix 1 describes each instrument, the formula used to calculate the score, and the interpretation of the scores generated by each instrument. To be a Grade 6 level and under, the scores for the FKG needed to be 6 or lower. FRE scores ranged from 0 to 100, with a higher score corresponding to a text that is easier to read (Table 1). An FRE score between 60 and 70 corresponded to a standard reading level. The online calculator, Readable, was used by other peer-reviewed publications, and we used the validated readability formulas in Table 1 [21,22]. The FRE and FKG scores have been used to evaluate medical literature and are the most applicable readability formulas for health information [18,23-27].

**Table 1:** Flesch-Kincaid reading ease score interpretation.

Score	Interpretation
90 to 100	Very easy
80 to <90	Easy
70 to <80	Fairly easy
60 to <70	Standard
50 to <60	Fairly difficulty
30 to <50	Difficult
0 to <30	Very difficult

#### Quality of Patient Education Material

DISCERN is a tool designed for patients and health care providers to assess the reliability and quality of written material on treatment choices without the need for medical knowledge [28]. It is a 16-question survey that covers the reliability of a publication, treatment options, benefits, and risks of treatment options. Table 2 describes the interpretation of the total DISCERN scores. A higher score indicates a higher quality of the publications. The DISCERN scores were independently performed by a senior medical student who was trained in using the DISCERN tool. The DISCERN score has been used by other senior medical students in other peer-reviewed publications [29-31].

**Table 2:** DISCERN scores.

Score range	Quality rating
63-80	Excellent
51-62	Good
39-50	Fair
27-38	Poor
16-26	Very poor

### Statistical Analyses

Categorical variables were reported using frequencies and proportions. Continuous variables are presented as means (SD) or medians and interquartile ranges. Separate analyses were conducted to determine if quality and readability differed depending on the origin of the PEM. These were compared using the Kruskal Wallis test, followed by the Dunn-Bonferroni post hoc tests. The Spearman correlation coefficients were used to assess the relationship between DISCERN scores and readability scores. Statistical analyses were performed using SPSS, version 26.0 (IBM Corp), with statistical significance set to *P*<.05.

## Results

### Search Results

A total of 80 webpages were retrieved from the search. After the removal of 4 duplicates and excluding 35 webpages, 41 webpages met the inclusion criteria. Moreover, 63% of the webpages originated from the United States (26/41) while 37% (15/41) originated from Canada. Of the included webpages, 2% (1/41) were from an academic institution, 17% (7/41) from a professional organization, 36% (15/41) were from a medical information website, 17% (7/41) were from a government website, 21% (9/41) were from private clinics, and 4% (2/41) were from miscellaneous websites. Of the excluded webpages, 2% (1/35) were from a blog, 17% (6/35) were from a scientific webpage, 25% (9/35) were from websites targeting medical professionals, and 45% (16/35) were websites without patient information pertaining to diabetic wound care.

### Readability Evaluation

The FRE scores ranged from 0 to 100 with a higher score corresponding to a text that is easier to read (Table 1). An FRE score between 60 and 70 corresponds to a standard reading level. The mean FRE score for all included PEM was 63.43 (SD 14.21), indicating a standard difficulty with a range of 33.8-84.2. Moreover, 68% (58/85) had FRE scores below 60, indicating that they were “fairly difficult” to “very difficult” to read. The mean reading grade levels as determined by the FKG score was 7.85 (SD 2.38). When looking at PEM from different origins, PEM from government websites had the highest FRE scores. PEM from private clinics had the highest FKG scores (Table 3). PEM from the United States also appeared to have a higher reading level than Canadian PEM (Table 4).

**Table 3:** Mean readability scores according to each type of website (95% CI).

	Academic institutions (n=1)	Private clinics (n=15)	Professional organizations (n=7)	Medical information websites (n=7)	Government websites (n=9)	Miscellaneous (n=2)	Total (N=41)
FRE^a^ score (SD)	71.9 (—^b^)	55.4 (8.4)	68.9 (7.4)	61.69 (8.4)	73.19 (5.17)	55.1 (21.9)	7.85 (2.38)
FKG^c^ score (SD)	6.7 (—)	8.63 (1.3)	7.54 (1.8)	8.15 (1.4)	6.46 (1.1)	8.6 (3.9)	63.43 (14.21)

^a^FRE: Flesch-Kincaid reading ease.

^b^Not applicable.

^c^FKG: Flesch-Kincaid grade level.

**Table 4:** Mean readability scores according to country of origin (95% CI).

	Canada	The United States
FRE^a^ score (SD)	66.52 (6.7)	55.4 (5.7)
FKG^b^ score (SD)	7.67 (1.1)	8.63 (1.0)

^a^FRE: Flesch-Kincaid reading ease.

^b^FKG: Flesch-Kincaid grade level.

### Quality of Patient Education Material

The mean DISCERN score was 45.66 (SD 3.34) (Table 5). The weighted κ statistic for the total DISCERN scores was 0.95. The average scores for each item in the DISCERN instrument are displayed in Multimedia Appendix 1. Twenty-seven percent (11/41) of articles had total DISCERN scores of less than 39, indicating they were of “poor” or “very poor” quality. Table 6 demonstrates the DISCERN scores for the PEM based on their origin. PEM originating from medical information websites had significantly higher DISCERN scores (*P*=.01). There was no significant correlation between DISCERN score and FRE score (*r*=0.07, *P*=.67) or DISCERN score and the average reading grade level (*r*=-0.005, *P*=.97).

**Table 5:** Average score (95% CI) for each item in the DISCERN instrument.

Quality criterion		Value
**Section 1: reliability, mean (SD)**		
	Are the aims clear?	3.1 (0.3)
	Does it achieve its aims?	4.1 (0.3)
	Is it relevant?	3.7 (0.3)
	Is it clear what sources of information were used to compile the publication (other than the author or producer)?	2.6 (0.4)
	Is it clear when the information used or reported in the publication was produced?	2.6 (0.5)
	Is it balanced and unbiased?	2.7 (0.3)
	Does it provide details of additional sources of support and information?	2.6 (0.3)
	Does it refer to areas of uncertainty?	2.0 (0.4)
Total reliability score, mean (SD)		23.4 (1.8)
**Section 2: quality, mean (SD)**		
	Does it describe how each treatment works?	3.4 (0.4)
	Does it describe the benefits of each treatment?	2.8 (0.4)
	Does it describe the risks of each treatment?	3.0 (0.9)
	Does it describe what would happen if no treatment were used?	3.1 (0.4)
	Does it describe how the treatment choices affect overall quality of life?	2.5 (0.3)
	Is it clear that there may be more than one possible treatment choice?	3.1 (0.3)
	Does it provide support for shared decision-making?	3.0 (0.4)
Total quality score, mean (SD)		19.2 (1.6)
Overall rating of sites, mean (SD)		3.0 (0.3)
Total DISCERN scores, mean (SD)		45.7 (3.3)

**Table 6:** Mean DISCERN score for patient education materials based on their origin.

PEM^a^ origin	Value
Academic institutions, mean (SD)	42.00 (—^b^)
Professional organizations, mean (SD)	41.57 (9.52)
Medical information websites, mean (SD)	53.53 (5.99)
Government websites, mean (SD)	42.43 (5.26)
Private clinics, mean (SD)	39.56 (15.83)
Miscellaneous, mean (SD)	41.50 (6.36)

^a^PEM: patient education materials.

^b^Not applicable.

## Discussion

### Principal Findings

On average, PEM on diabetic foot ulcer care were written at an approximate reading level of grades 7-8, which exceeds the 6th-grade reading level recommendation from the Canadian Medical Protective Association and the average reading level of a North American adult [14]. Furthermore, 68% had FRE scores below 60, indicating that they were “fairly difficult” to “very difficult” to read. Similar results have been found by other studies. Lipari et al conducted a study on the readability of online PEM on diabetes mellitus and found that 77% of PEM (10/13) were written above an 8th-grade reading level [14]. Furthermore, PEM from Diabetes Canada were written at about a 7th-10th grade reading level. This is an important finding as some may assume that material originating from credible sources such as academic institutions and professional organizations may be better for patient education. Our study found that PEM from professional organizations and an academic institution typically exceeded a 6th-grade reading level. This is in keeping with a previous study, which found that PEM on diabetes mellitus from US academic institutions and professional organizations were written for a reading level of above grade 10 [32].

PEM must also be reliable, comprehensive, and contain evidence-based information. This study attempted to assess reliability and quality through the DISCERN tool. Twenty-seven percent (11/41) of the articles had total DISCERN scores less than 39, indicating they were of “poor” or “very poor” quality. Similar studies on diabetic retinopathy have found that 73% (16/22) of PEM were of “poor” or “very poor” quality [33]. Interestingly, this study also found that academic institution and medical information websites had significantly higher reliability scores when compared with private clinics. These differences may be because academic institutions and medical information websites have access to several experts in their respective fields and may have more resources to produce more robust PEM. These findings may have important implications when physicians and other allied health professionals refer patients to online resources to learn more about diabetic foot ulcers.

This study performed a correlation analysis to determine the relationship between DISCERN scores and readability. A weak positive correlation was found between DISCERN scores and FRE scores, and a weak negative correlation was found between DISCERN scores and average reading grade level. Neither reached statistical significance. This implies that high-quality, more reliable PEM were not necessarily more readable. While the target audiences for these websites vary, these were the websites most readily accessible and targeted to patients. This has an important implication as easily accessible PEM that patients can easily comprehend may not necessarily be of high quality.

### Limitations

This study has several limitations. Firstly, the search strategy in this study used the Google search engine with 4 different search terms to appropriately simulate how patients search the internet for health information. It is possible that patients could obtain different resources. However, Google is the most common search engine used and has been the sole search engine used in several other readability analyses [14,33,34]. Furthermore, it is not possible to predict which search terms patients will use. However, this study utilized 4 different search terms that are the most likely terms used by patients searching for diabetic foot ulcer care. We did not account for patients who do not use the internet to access PEM on diabetic foot ulcers or those who do not have access to a computer.

The correlation between readability scores and true reading comprehension cannot be considered perfect since readability scores have several limitations. Since these scores are based on variables such as the number of syllables or characters per word, they can be skewed by medical terminology such as “vasculature” or “neuropathy.” Titles and headings can also mislead them as these may be interpreted as sentences. This study mitigates these limitations by using readability formulas most suited for medical literature and appropriately preparing the text from websites. It is important to note that readability scores are not measures of overall comprehension. Rather, readability scores reflect one of the many characteristics of reading skill and reading ease of materials [35,36]. Some suggestions for improving the readability of PEM include minimizing the use of complex words and decreasing the length of sentences or syllables per word. Readability scores should be considered with other indicators in assessing the overall comprehension of written PEM. Our study attempts to address this by using readability scores along with the DISCERN tool. Lastly, although the DISCERN tool has been validated and widely applied to patient information on treatment options, it does not directly evaluate the accuracy of the information contained within these PEM. Rather, DISCERN determines the readability and quality of public materials.

### Conclusion

As the COVID-19 pandemic has placed a greater emphasis on digital health, it is important to assess the readability and quality of online information of DFU to ensure adequate and appropriate patient education. While the internet has allowed for ease of access to information for a breadth of patients, our study showed that online PEM on DFU care were far above the recommended reading level for patients. Physicians and other allied health professionals should be aware of the deficiencies in the quality and reliability of internet-based PEM that patients use to inform their care. In the future, PEM authors should consider using these tools to evaluate the readability and quality of their website.

## Appendix

Multimedia Appendix 1 Instruments and calculations used to assess readability.

## Abbreviations

DFU: diabetic foot ulcers
FKG: Flesch-Kincaid grade level
FRE: Flesch-Kincaid reading ease
PEM: patient education materials
