# Supplementary material for: Internet-Based Patient Education Materials Regarding Diabetic Foot Ulcers: Readability and Quality Assessment
Source: JMIR Diabetes. 2022 Jan 11;7(1):e27221. doi: 10.2196/27221 (PMC8790680; doi:10.2196/27221)
Supplement: Multimedia Appendix 1 [file diabetes_v7i1e27221_app1.docx]

Multimedia Appendix 1**:** Instruments and calculations used to assess readability

| Assessment Scale | Formula | Interpretation of Score |
| --- | --- | --- |
| FRE | FRE = 206.835 − (84.6 × average # of syllables per word) − (1.015 × average # of words per sentence) | Determines readability using a 0-100 score, with higher scores indicating a higher level of readability [38].The optimal score for the FRE is 65 [39]. |
| FKG | FKG = (11.8 × average # of syllables per word) + (0.39 × average # of words per sentence)− 15.59 | Indicates reading level that can be comprehended by a person who has graduated from that grade level [40]. |

FRE: Flesch-Kincaid Reading Ease, FKG: Flesch Kincaid Grade Level
